# 3D Printing for Pelvic Organ Prolapse Management: A Narrative Review of Emerging Applications

**DOI:** 10.3390/bioengineering13050488

**Published:** 2026-04-23

**Authors:** Xinyi Wei, Xiaolong Wang, Xin Yang, Mingjing Qiao, Yannan Chen, Andre Hoerning, Xianhu Liu, Chenchen Ren

**Affiliations:** 1 Tianjian Laboratory of Advanced Biomedical Sciences, Department of Gynecology, the Third Affiliated Hospital of Zhengzhou University, Zhengzhou University, Zhengzhou 450052, China; weixinyi235@gmail.com (X.W.);; 2 National Clinical Research Center for Obstetrics and Gynecology, Henan Branch, Zhengzhou 450052, China; 3 Department of Pediatrics and Adolescent Medicine, University Hospital Erlangen, Friedrich-Alexander-University Erlangen-Nürnberg, 91054 Erlangen, Germany; 4 Key Laboratory of Material Processing and Mold of Ministry of Education, National Engineering Research Center for Advanced Polymer Processing Technology, Zhengzhou University, 97-1 Wenhua Road, Zhengzhou 450002, China

**Keywords:** 3D printing, pelvic organ prolapse, urogynecology, surgical mesh, patient-specific implant

## Abstract

Pelvic organ prolapse (POP) is a common benign gynecological disorder that substantially affects quality of life, particularly in aging female populations. Current management strategies, including standardized vaginal pessaries and synthetic surgical meshes, are often limited by poor anatomical adaptability, mechanical mismatch with native pelvic tissues, and long-term safety concerns. These limitations have driven increasing interest in personalized and biomechanically compatible therapeutic solutions. Three-dimensional (3D) printing, also known as additive manufacturing, has emerged as a promising bioengineering technology to address these unmet clinical needs. By enabling layer-by-layer fabrication directly from digital models, 3D printing allows for precise control over device geometry, mechanical properties, and material composition, facilitating patient-specific design. This narrative review summarizes recent progress in 3D printing for POP management across three major application domains: (i) next-generation meshes based on biodegradable polymers, elastomeric materials, natural biomaterials, and hydrogel systems; (ii) customized vaginal pessaries tailored to individual pelvic anatomy using imaging-assisted workflows; and (iii) imaging-based pelvic models and prototype devices for surgical planning, education, and exploratory assessment. Overall, existing studies demonstrate that 3D printing enables improved biomechanical compatibility, enhanced tissue integration, and multifunctional device design, including drug delivery capability. Although current evidence is largely pre-clinical or based on pilot studies, additive manufacturing holds strong potential to advance POP management toward safer, personalized, and functionally optimized clinical solutions.

## 1. Introduction

Pelvic organ prolapse (POP) is a benign gynecological disorder characterized by the descent of pelvic structures, which may manifest as a vaginal bulge or a sense of pelvic pressure [1]. Affected women often report urinary disturbances, bowel dysfunction, and sexual difficulties, all of which can markedly diminish daily functioning and overall quality of life [2,3,4]. Population-based research indicates that nearly one in eight women in the United States is expected to require surgical treatment for POP at some point in her life [5]. While younger women can also be affected, the highest burden of symptomatic POP is typically reported among those in their seventies [6]. As the population continues to age, projections suggest that the prevalence of POP could increase by about 50% by the year 2050 [7]. Given this growing clinical and societal burden, effective management strategies are of increasing importance [8].

A range of therapeutic approaches has been developed, spanning conservative to surgical intervention [9]. Conservative management typically includes lifestyle modifications and pelvic floor muscle training, which are generally recommended for women with mild symptoms. Vaginal pessaries, as a commonly used conservative device, can provide symptom relief and are particularly suited for elderly patients or those who are unsuitable for surgery [8].

Surgical management includes native tissue repair, sacrocolpopexy, and the implantation of synthetic or biological meshes to reinforce weakened pelvic tissues [10]. Mesh implants were once widely adopted to lower recurrence rates compared with native tissue repair [11]. However, serious complications such as erosion, infection, chronic pain, and dyspareunia raised major safety concerns. In April 2019, the U.S. Food and Drug Administration (FDA) mandated the withdrawal of all transvaginal mesh devices intended for POP repair from the U.S. market, concluding that manufacturers had not provided adequate data to demonstrate durable safety and efficacy compared with native tissue repair [12,13]. Beyond these complications, a fundamental drawback of currently available meshes is their lack of customization, which limits anatomical fit and patient-specific adaptation [14].

These limitations, shared by both vaginal pessaries and surgical meshes, highlight the unmet need for safer, more effective, and patient-tailored therapeutic options in POP management.

These limitations underscore the necessity of exploring innovative technologies capable of offering individualized and safer solutions. Three-dimensional (3D) printing, also referred to as additive manufacturing, has recently emerged as a promising tool in this regard. Unlike conventional manufacturing methods, 3D printing builds objects layer by layer directly from digital models, enabling the fabrication of highly complex geometries without the need for molds [15]. This characteristic makes it particularly suitable for addressing anatomical variability among women with POP, where device size, shape, and mechanical properties must often be tailored to individual needs. By integrating imaging data such as ultrasound, computed tomography (CT), or magnetic resonance imaging (MRI) into computer-aided design, 3D printing enables the creation of personalized devices with precise anatomical conformity. Flaxman et al. demonstrated a workflow for generating patient-specific gynecologic models from MRI data using 3D printing, highlighting its potential for individualized applications [16]. Moreover, Nguyen et al. confirmed that patient-specific 3D-printed models achieve high dimensional accuracy across multiple printing technologies, with deviations typically within a few millimeters, underscoring their clinical reliability [17]. Another significant advantage of 3D printing lies in its material versatility. A notable advantage of 3D printing lies in its material versatility. Biodegradable polymers such as polycaprolactone (PCL) and polylactic acid (PLA) can be readily processed, offering both adequate mechanical support and favorable tissue integration for pelvic floor repair [18]. Furthermore, renewable natural fibers and biopolymers like cellulose, alginate, collagen, silk, and gelatin have emerged as sustainable materials in biomedical 3D printing. These bio-derived components can enhance biodegradability, mimic extracellular matrix properties, and improve scaffold compatibility [19]. In addition to patient-specific implants, 3D printing can also generate pelvic models that facilitate surgical planning and medical education [20].

**Figure 1:**
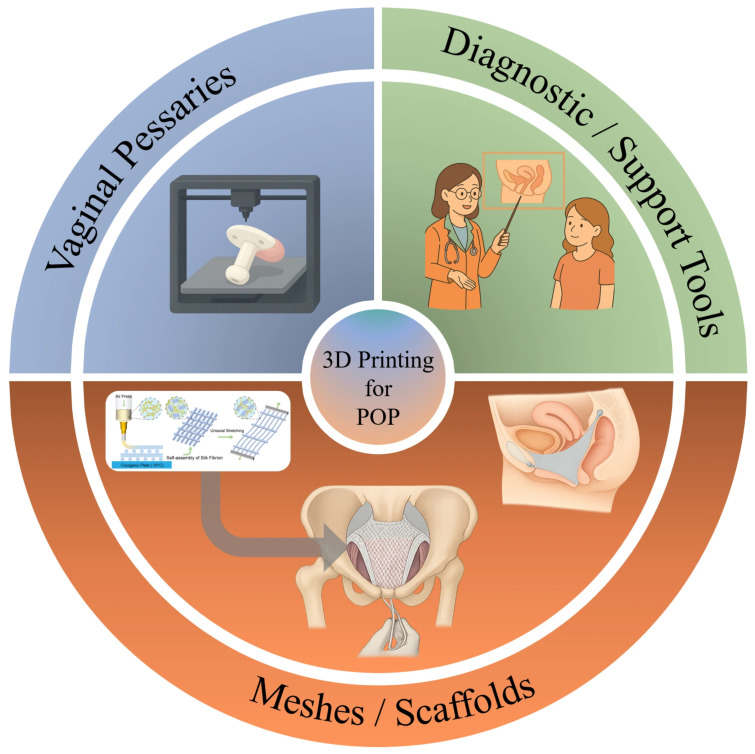
A graphical abstract illustrating the scope of this review. Three-dimensional (3D) printing offers new opportunities in the management of POP.

Despite the growing interest in pelvic floor disorders and advances in surgical and conservative management, comprehensive discussions specifically addressing the role of 3D printing in POP remain scarce. Most existing reports are limited to pilot studies or material-focused investigations, and an integrative overview of its clinical potential is still lacking. Therefore, as shown in Figure 1, this review aims to provide a focused summary of current progress in 3D printing for POP management. We highlight three major application domains: (1) 3D-printed meshes with improved biocompatibility and drug delivery potential, (2) patient-specific vaginal pessaries tailored to individual anatomy, and (3) imaging-assisted pelvic models for surgical planning and medical education. By outlining these directions, we seek to underline both the present opportunities and the prospects of this emerging technology in urogynecology.

## 2. Applications of 3D Printing in POP

**Figure 2:**
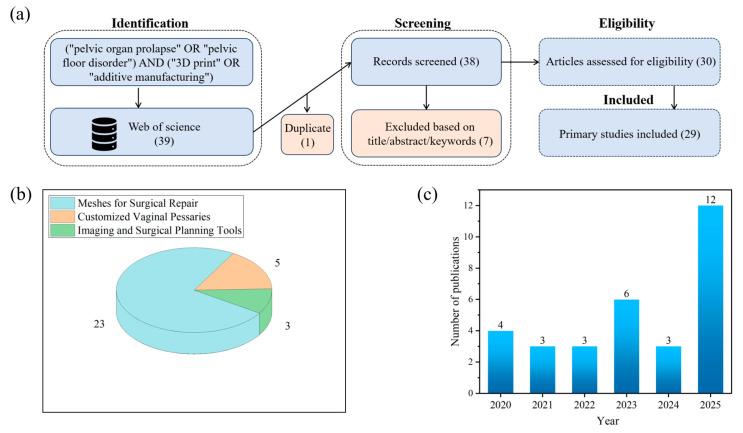
An overview of the literature selection and distribution. (**a**) PRISMA-based flow diagram of the literature screening process. (**b**) The distribution of included studies across three major application areas of 3D printing in pelvic organ prolapse: meshes for surgical repair (23), customized vaginal pessaries (5), and imaging/surgical planning tools (3). (**c**) The annual trend in the number of publications from 2020 to 2025, showing a marked increase in recent years.

To provide a transparent and structured overview of current research on the applications of three-dimensional (3D) printing in POP, this narrative review adopted a systematic-style literature search strategy. Specifically, we searched the Web of Science database using the following keywords: “pelvic organ prolapse” OR “pelvic floor disorder” AND “3D print” OR “additive manufacturing”. As illustrated in Figure 2a, a total of 39 records were initially retrieved. After removal of duplicates and screening of titles, abstracts, and keywords, 31 primary studies were identified and included for full-text analysis.

As shown in Figure 2b, the distribution of these studies highlights three main areas of application. The vast majority (23/31) focused on the development of surgical meshes, reflecting the urgent need for safer, more biocompatible, and customizable alternatives to conventional polypropylene implants. In contrast, a smaller yet growing body of work (5/31) explored the customization of vaginal pessaries, aiming to provide personalized conservative treatment options. Another 3 studies investigated imaging-based diagnostic and surgical planning tools, which represent emerging innovations toward individualized assessment and preoperative decision-making.

The temporal distribution of publications further demonstrates a surge of research interest in this field, with a marked increase observed between 2023 and 2025. Together, these findings underscore the growing momentum of 3D printing technologies in urogynecology. In the following sections, we will discuss each of the three domains in detail: surgical meshes (Section 2.1), customized vaginal pessaries (Section 2.2), and imaging or surgical planning tools (Section 2.3).

### 2.1. Meshes for Surgical Repair

#### 2.1.1. Clinical Needs and Limitations of Conventional Meshes

POP is traditionally treated by reinforcing weakened pelvic tissues with surgical meshes. For decades, non-degradable polypropylene (PP) meshes have been the mainstay, offering immediate mechanical support and reducing recurrence compared to native tissue repair. However, long-term clinical follow-ups have revealed high complication rates, including mesh erosion, chronic pain, dyspareunia, infection, and shrinkage, which eventually led to regulatory restrictions and even bans on transvaginal PP meshes in many countries [21,22,23]. A fundamental drawback of conventional meshes is their stiffness mismatch and limited capacity for biological integration, resulting in foreign body reactions and poor patient outcomes [24]. Although traditional polypropylene meshes have provided durable support in selected settings, their complication profile has substantially limited their use, especially in transvaginal POP repair, and has motivated the development of next-generation 3D-printed mesh systems.

In response to these limitations, the clinical requirements for POP repair meshes have undergone a fundamental shift: an ideal implant should exhibit mechanical properties closely matching those of native pelvic tissues while providing sufficient long-term support, possess a microarchitecture that facilitates tissue integration and vascularization, demonstrate excellent biocompatibility with minimal chronic inflammation, and allow a degree of customization to accommodate interindividual anatomical variability and different prolapse compartments [25]. The future direction of POP mesh development is moving away from permanent inert scaffolds toward biologically functional constructs that support coordinated tissue remodeling and regeneration.

Recent advances in 3D printing provide opportunities to design patient-specific, mechanically compliant, and multifunctional meshes, potentially overcoming the shortcomings of traditional devices [26]. In this section, the applications of 3D printing in the fabrication of meshes for POP repair are introduced, categorized according to the types of materials employed. This organization highlights the distinctive properties and advantages of different material systems in the context of POP treatment.

#### 2.1.2. Material Innovations for 3D-Printed Meshes

##### Polycaprolactone (PCL)

Polycaprolactone (PCL) is one of the most widely investigated polymers for tissue engineering and pelvic floor reconstruction, owing to its unique combination of biodegradability, biocompatibility, and mechanical tunability [27]. As an aliphatic polyester, PCL undergoes slow hydrolytic degradation, typically over 2–3 years, which ensures that it provides prolonged mechanical support during the critical phase of tissue ingrowth and remodeling [28]. Its relatively low melting point and high processability make it particularly well suited for additive manufacturing techniques, including fused deposition modeling and melt electrowriting, which allow for the fabrication of scaffolds with finely controlled architectures. Moreover, PCL has been approved by the FDA for certain biomedical applications [29], further underscoring its safety profile and translational potential. Compared with conventional polypropylene meshes, PCL-based scaffolds offer improved compliance and reduced risk of long-term complications, making it a promising candidate for next-generation degradable implants in pelvic organ prolapse repair.

**Figure 3:**
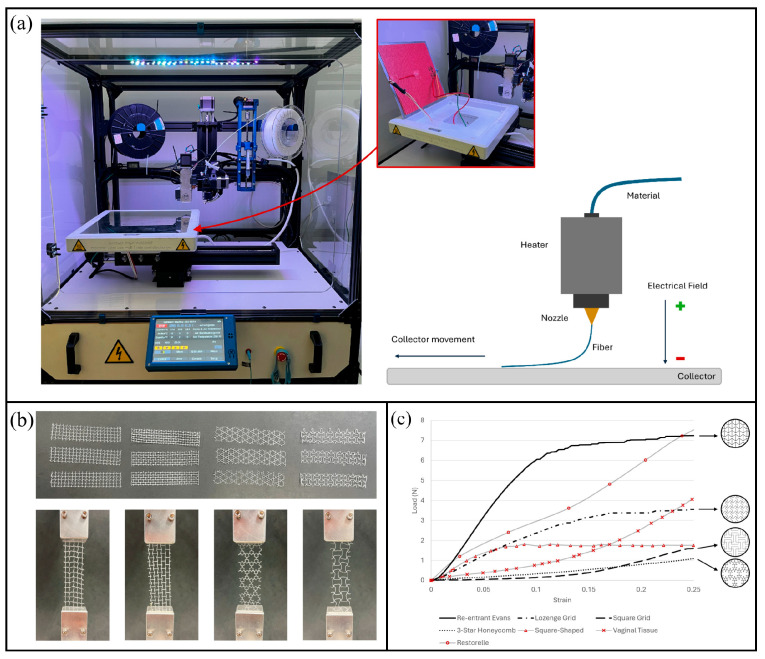
(**a**) Modified melt electrowriting (MEW) setup with a heated print bed, improving filament fusion and interlayer adhesion for stable mesh fabrication. (**b**) Four auxetic geometries—re-entrant Evans, lozenge grid, three-star honeycomb, and square grid—subjected to uniaxial tensile testing, highlighting structural versatility and biomechanical tuning potential. (**c**) Force–strain comparison between PCL auxetic meshes, native vaginal tissue, and commercial polypropylene meshes [28].

Melt electrowriting (MEW) is a high-precision additive manufacturing technique that enables the controlled deposition of micro-scale polymer fibers, producing scaffolds with well-defined pore architectures and mechanical stability [30]. In the recent study by Ferreira et al., the MEW system was modified with a heated print bed to improve filament fusion and interlayer adhesion [31]. Figure 3a illustrates this setup, which ensures the uniformity and robustness of the printed meshes.

The authors fabricated four auxetic geometries—re-entrant Evans, lozenge grid, three-star honeycomb, and square grid—to investigate their biomechanical compatibility with vaginal tissue. Figure 3b shows these configurations under tensile testing. Among them, the re-entrant Evans design displayed excessive stiffness due to overlapping struts, while the lozenge grid offered an intermediate mechanical profile. In contrast, the square grid and three-star honeycomb geometries exhibited hyperplastic-like responses that most closely resembled vaginal tissue behavior, making them the most promising candidates for pelvic floor repair.

While Figure 3b highlights structural variability, Figure 3c further demonstrates how these geometries perform under mechanical loading compared with biological tissues and conventional meshes. A key observation is the avoidance of the so-called ‘bundling effect’, a phenomenon in knitted or woven meshes where fibers progressively engage under tension, leading to localized stiffening and uneven stress distribution [32]. This effect has been linked to tissue erosion and chronic inflammation in polypropylene meshes [25]. By distributing stress more evenly, the square grid and three-star honeycomb mesh achieved better biomechanical compatibility, underscoring the potential PCL as a foundation for next-generation degradable implants.

Beyond the work of Ferreira et al., a series of investigations have highlighted how the micro-architecture of PCL meshes can be tuned through melt electrowriting (MEW) and related extrusion-based techniques to better reproduce the mechanical environment of the pelvic floor.

Da Cunha et al. developed a custom MEW platform to fabricate PCL meshes with fiber diameters in the range of around 80–100 μm [33]. By systematically varying fiber alignment and pore geometry, they demonstrated that printed constructs exhibited elastic behavior within the physiological deformation range, closely approximating the compliance of vaginal tissue. Compared with the commercial polypropylene mesh, which was significantly stiffer, the MEW-printed PCL meshes displayed stress–strain curves more compatible with native tissue, underscoring their translational potential.

In a complementary study, Rynkevic et al. examined five different configurations of PCL meshes, including fiber diameters of 240 μm, 160 μm, and multiple layers of 80 μm fibers [34]. Their degradation experiments over 90–180 days revealed that thinner fibers (80 μm) degraded more rapidly and exhibited weight loss up to 27% under acidic conditions, while thicker fibers retained structural integrity for longer durations. Mechanical testing confirmed that multilayer constructs offered improved compliance compared to thicker single-layer designs, highlighting the importance of fine control over fiber size and deposition strategy.

Sterk et al. exploited the design freedom of 3D printing to fabricate sinusoidal and auxetic geometries using PCL [35]. They showed that identical polymer materials could achieve widely different Young’s modulus values (ranging from ~50 to 400 N/mm^2^) depending on the geometry, and that auxetic designs maintained dimensionally stable pores under tensile load. This is of particular importance because pore collapse and stiffening are major shortcomings of conventional knitted meshes.

More recently, Vaz et al. compared non-medical and medical grade PCL printed by MEW using a pellet extruder [36]. Meshes were produced with filament diameters of 80, 160, and 240 μm in both square and diagonal configurations. Mechanical testing against sheep vaginal tissue revealed that the commercial polypropylene mesh was approximately 84% stiffer at higher strain levels, whereas MEW-printed PCL meshes with 80 μm filaments differed by less than 10% from tissue response. This precise tunability of stiffness and compliance suggests that MEW-printed PCL meshes could overcome the mechanical mismatch that has plagued polypropylene implants.

Together, these studies demonstrate that MEW enables microscale control of fiber diameter, pore geometry, and layering strategies, yielding degradable meshes with mechanical properties much closer to those of vaginal tissue than commercial alternatives. The ability to fine-tune stiffness, elasticity, and degradation profiles at the design stage is a defining advantage of PCL-based constructs.

In addition to structural optimization, PCL also serves as a versatile platform for composite and functionalized meshes. Its high processability enables blending with hydrophilic polymers, natural proteins, or inorganic fillers to enhance elasticity, bioactivity, or drug delivery capacity [37]. For instance, silk fibroin has been incorporated to improve cell adhesion [38], while nano-hydroxyapatite has been applied to increase stiffness and enable sustained antibacterial release [39]. Ren et al. also demonstrated the feasibility of PEG-modified PCL meshes with antimicrobial coatings [40]. Although the detailed outcomes of these approaches are discussed in the following section on functionalization, these examples highlight the adaptability of PCL as a base polymer for multifunctional implants.

Importantly, preliminary in vivo studies have reinforced the translational potential of PCL meshes. Pilot animal experiments in rodent studies [40] have shown superior tissue integration and reduced inflammatory responses compared with polypropylene meshes. Such findings emphasize that beyond favorable in vitro mechanics, PCL meshes also induce a more compatible host response, a prerequisite for clinical application.

Taken together, PCL has emerged as the most extensively investigated biodegradable polymer for pelvic organ prolapse repair. Its advantages lie not only in its biodegradability and biocompatibility, but also in the design flexibility enabled by advanced 3D printing methods. By enabling precise tuning of stiffness, pore geometry, and degradation profiles, PCL meshes can approximate the mechanical performance of native vaginal tissue far better than polypropylene. At the same time, its potential for composite functionalization and promising preclinical data provide a robust foundation for translation. Nevertheless, the relatively low elasticity of PCL compared with soft pelvic tissues suggests the need to explore more elastic alternatives, such as thermoplastic polyurethane (TPU), which will be discussed in the next section.

##### Thermoplastic Polyurethanes

Thermoplastic polyurethanes (TPUs), including conventional TPU and the more advanced polycarbonate-urethane (PCU), have recently emerged as attractive alternatives to polypropylene (PP) for POP meshes. These polymers are segmented block copolymers composed of alternating “soft” and “hard” segments, giving them a unique combination of elasticity, toughness, and processability [41]. Compared to rigid PP, TPU-based meshes are softer, more compliant, and exhibit fatigue resistance closer to that of vaginal tissue, which is essential to minimize erosion and chronic pain.

Despite these shared features, conventional TPU and PCU differ in their chemical backbones and, consequently, in long-term performance. TPU, typically based on polyester or polyether soft segments, is flexible and easily processable, although certain formulations may be susceptible to hydrolysis and oxidative degradation during prolonged implantation [42]. PCU, in contrast, incorporates polycarbonate-based soft segments, providing superior hydrolytic and oxidative stability, higher fatigue resistance, and improved biocompatibility, making it more suitable for long-term implant [43], thus reducing the risk of chronic inflammatory response and mechanical failure in long-term use. These characteristics explain why PCU has been widely investigated for blood-contacting devices and permanent biomedical implants, while TPU has attracted attention in the development of flexible, drug-loaded scaffolds for soft tissue reinforcement.

Both materials are compatible with additive manufacturing techniques such as fused deposition modeling (FDM), allowing for the fabrication of customized meshes with tailored architecture [44,45]. This compatibility not only enables patient-specific design but also facilitates the incorporation of bioactive components during fabrication. Their inherent elasticity, tissue-mimicking compliance, and ability to integrate functional agents (such as antibiotics [46] or hormones [47]) position TPU and PCU as promising candidates for next-generation POP repair meshes.

**Figure 4:**
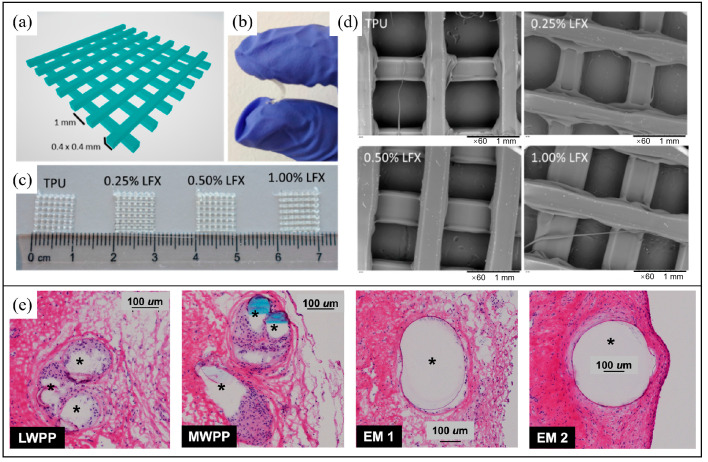
A CAD 3D image of the two layer meshes with its dimensions (**a**). Representative image showing the flexibility of a TPU-based mesh (**b**). Image of TPU and TPU loaded with LFX 3D printed meshes (**c**). SEM images of TPU and LFX loaded TPU 3D printed meshes (**d**) [48]. (**e**) Representative hematoxylin and eosin (H&E)-stained histological sections showing host cellular responses to implanted meshes in a rabbit vaginal model, as reported by Knight et al., Compared to lightweight (LWPP) and midweight (MWPP) polypropylene meshes, 3D-printed polycarbonate urethane elastomeric membranes (EM 1 and EM 2; 0.4 mm × 0.4 mm struts) induced reduced inflammatory cell accumulation around the fibers (asterisks), despite a substantially higher mesh weight [50].

Domínguez-Robles et al. developed antibacterial TPU-based meshes using hot-melt extrusion (HME) followed by FDM, aiming to overcome the mechanical mismatch and infection risk associated with polypropylene (PP) meshes in pelvic organ prolapse repair27. The authors first designed a two-layer grid architecture (Figure 4a) and printed it using TPU filaments loaded with varying concentrations of levofloxacin (LFX). The resulting meshes exhibited high flexibility and deformability (Figure 4b), which is critical for mimicking the compliance of vaginal tissue and avoiding complications such as erosion or chronic pain. Visual inspection of the printed samples (Figure 4c) showed no macroscopic differences between drug-free and drug-loaded meshes, suggesting homogenous incorporation of the antibiotic. Scanning electron microscopy further confirmed this uniformity, revealing smooth filament surfaces without detectable drug aggregation or surface crystallization across all formulations (Figure 4d). These results demonstrate the feasibility of producing structurally robust, drug-eluting meshes via FDM with potential for patient-specific customization and early postoperative infection control. Knight et al. (2024) conducted an in vivo comparative study evaluating 3D-printed PCU meshes versus conventional polypropylene meshes (PPMs) for pelvic floor repair, focusing on mechanical tuning and host tissue integration [48]. As illustrated in Figure 4e, immunofluorescent micrographs show the macrophage response at 12 weeks post implantation in a rabbit vaginal model. Two PCU elastomeric membrane (EM) designs (EM 1 and EM 2), differing in stiffness but sharing similar pore size and fiber geometry (0.6 mm × 0.4 mm), were compared with lightweight (LW) and midweight (MW) PPMs. Macrophages (RbM2^+^, green) accumulated densely around PPMs, forming disorganized, multilayered inflammatory capsules. In contrast, both PCU-based constructions elicited a markedly attenuated immune response, characterized by thin, monolayered macrophage layers and better preservation of native tissue architecture. Quantitative analysis revealed lower total macrophage density and macrophage-to-cell ratios in PCU groups, despite their higher mesh weight (up to 169 g/m^2^), suggesting that material stiffness and compliance, rather than absolute weight, were key determinants of biocompatibility. These findings support the use of soft, viscoelastic PCU materials for reducing chronic inflammation and improving host integration in next-generation POP meshes.

Building on these advances, several additional studies have further highlighted the versatility of TPU and PCU for pelvic floor applications. Farmer et al. fabricated TPU-based meshes via HME and FDM incorporating 17β-estradiol, demonstrating homogeneous drug distribution, stable thermal behavior, and sustained release over two weeks without compromising mechanical compliance [49]. These findings underscore the feasibility of combining mechanical reinforcement with localized hormonal therapy. Complementing these developments, Bachtiar et al. systematically evaluated 3D-printed PCU membranes, confirming stable pore geometry, compliance closer to vaginal tissue, and fatigue resistance on par with commercial meshes. Together, these studies reinforce the notion that TPU and PCU, through their tunable mechanics and compatibility with additive manufacturing, represent promising candidates for next-generation POP meshes capable of integrating both structural and therapeutic functionalities [50].

In summary, TPU and PCU represent promising material platforms for next-generation POP meshes. Combined with additive manufacturing, these polymers enable safer, more effective, and customizable solutions for pelvic floor reconstruction.

##### Silk Fibroin

**Figure 5:**
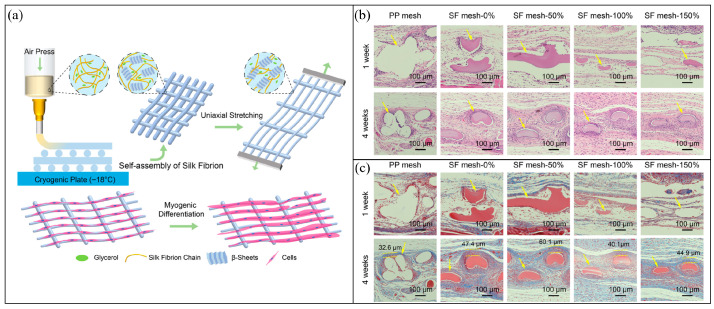
(**a**) The fabrication strategy of cryogenic 3D printing followed by post-stretching, generating ordered porous architectures with micro striped surfaces that guide directional cell growth. (**b**) H&E staining after implantation in a rat model shows reduced inflammatory infiltration and improved tissue integration with increasing stretching ratios compared to polypropylene (PP) mesh. (**c**) Masson trichrome staining reveals enhanced collagen deposition and more organized muscle fiber regeneration in SF meshes relative to PP controls [55].

Silk fibroin (SF), extracted from Bombyx mori cocoons, is a natural protein-based biomaterial that has gained increasing attention for biomedical use [51]. As a biodegradable and environmentally friendly material, SF undergoes enzymatic degradation into non-toxic amino acids that can be metabolized or reused by the body [52], avoiding the long-term complications associated with non-degradable PP and TPU meshes. In addition, SF exhibits excellent biocompatibility, supporting cell adhesion, proliferation, and extracellular matrix deposition, while eliciting only a mild immune response. Its outstanding mechanical strength, combined with tunable stiffness through modulation of β-sheet crystallinity, enables the fabrication of scaffolds with properties closer to soft tissues [53]. These unique features make silk fibroin a compelling candidate for POP meshes, where both mechanical compliance and regenerative capacity are required (here refer to Figure 5).

Wu et al. further explored the use of silk fibroin by fabricating composite meshes that combined SF with PCL through electrohydrodynamic printing [38]. The incorporation of SF not only enhanced the degradation profile of PCL but also improved cell affinity and soft tissue integration, as demonstrated in both vitro fibroblast assays and in vivo muscle defect models. Notably, the SF/PCL constructs promoted collagen fiber formation and muscle regeneration without eliciting severe inflammatory responses, validating their dual role as structural supports and bioactive scaffolds. Zheng et al. advanced this concept by developing cryogenically 3D-printed silk fibroin meshes followed by a post-stretching treatment [54]. As shown in Figure 5a, this strategy enabled the fabrication of highly ordered porous architectures with enhanced fibril alignment, generating micro striped surfaces that guided directional cell growth. Mechanical testing revealed that increasing the post-stretching ratio markedly improved tensile strength and elasticity, yielding constructs that better matched the compliance of vaginal tissues. Importantly, in vivo histological analyses further confirmed the biological advantages of these engineered meshes. Compared to polypropylene, silk fibroin constructs elicited reduced inflammatory infiltration, enhanced collagen deposition, and promoted the regeneration of organized muscle fibers (Figure 5b,c). Together, these findings highlight how structural engineering of silk fibroin meshes can simultaneously provide mechanical compliance and bioactive cues, underscoring their potential as regenerative implants for pelvic organ prolapse repair.

Collectively, these studies underscore the potential of silk fibroin, either alone or in hybrid formulations, to provide degradable, biocompatible, and mechanically adaptive alternatives to traditional synthetic meshes for POP repair.

On the other hand, emerging biomaterials and 3D printing strategies, including SF printing, have important drawbacks. Compared with conventional devices, they may show less certain long-term durability, more complex degradation behavior, greater manufacturing variability, and added challenges in sterilization and quality control. Moreover, patient-specific design and fabrication are likely to increase technical complexity, production time, and cost. As a result, the cost-effectiveness and scalability of these technologies remain to be established before routine clinical adoption can be justified.

##### Poly (Vinyl Alcohol) (PVA) Hydrogels

PVA is a synthetic, water-soluble polymer that has been extensively used in biomedical applications owing to its biocompatibility, hydrophilicity, tunable mechanics, and established safety record [56]. As a hydrogel-forming polymer, PVA is particularly attractive for POP meshes where a soft, compliant, and degradable scaffold is required to reduce mechanical mismatch with native tissues. Unlike rigid PP or even elastomeric TPU, PVA hydrogels can mimic the viscoelasticity of vaginal tissue, provide controlled swelling for nutrient transport, and be engineered with customizable degradation kinetics through chemical or physical cross-linking strategies [57].

**Figure 6:**
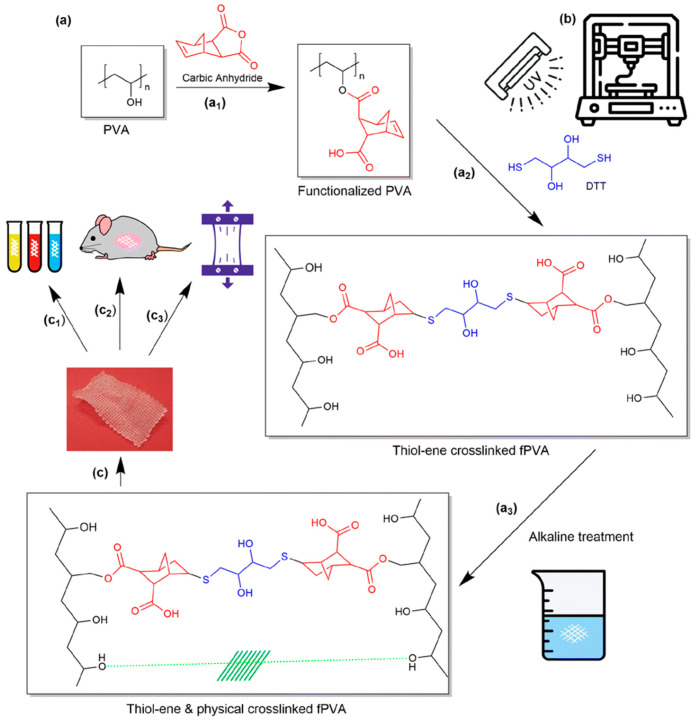
A schematic representation of programmable PVA cross-linking and 3D printing of tissue scaffolds. (**a**) Polymer chemistry: (a1) functionalization of PVA with carboxylic anhydride to yield fPVA (norbornene-modified), (a2) initial cross-linking via DTT reaction, and (a3) subsequent thiol–ene cross-linking and base-induced physical cross-linking; (**b**) UV-assisted DIW process enabling precise dimensional control; (**c**) characterization of fabricated scaffolds, including mechanical testing, thermal analysis (DSC/TGA), and cell viability/in vivo biocompatibility [58].

Chambers et al. exemplified this approach by fabricating dual-cross-linked PVA meshes through direct ink writing (DIW) [55]. As illustrated in Figure 6, the process in this work involves (a) the functionalization of PVA with norbornene groups followed by sequential cross-linking steps (thiolene chemistry and base-induced crystallite formation), (b) UV-assisted DIW to produce regular grid architectures with precise dimensional control, and (c) systematic evaluation of the resulting scaffolds, including mechanical, thermal, and biological properties. This strategy yielded meshes with a low and tunable elastic modulus (~4.9 MPa), closely approximating the compliance of vaginal tissue while maintaining structural robustness. Compared to commercial polypropylene-based meshes, the PVA constructs displayed more homogeneous deformation under tensile loading, moderate and controllable swelling ratios, and stable viscoelastic behavior in hydrated conditions. In vivo implantation in mice for 28 days confirmed minimal inflammation, limited fibrosis, and good tissue integration, supporting the potential of PVA hydrogels as safer, more adaptable alternatives for pelvic floor repair.

Overall, the study underscores that PVA hydrogels combine structural regularity, mechanical compliance, and biocompatibility, positioning them as promising candidates for next-generation POP meshes. While long-term degradation and fatigue performance remain to be evaluated, this work establishes hydrogel-based scaffolds as a complementary direction alongside PCL, TPU, and silk fibroin systems.

### 2.2. Customized Vaginal Pessaries

Vaginal pessaries have long served as a first-line, non-surgical management option for POP, particularly in women who are poor surgical candidates or prefer conservative therapy [59]. Traditional devices such as ring or Gellhorn pessaries are available in standardized sizes, but their effectiveness is often limited by anatomical variability, leading to discomfort, erosion, or poor retention. These shortcomings highlight the need for individualized solutions.

**Figure 7:**
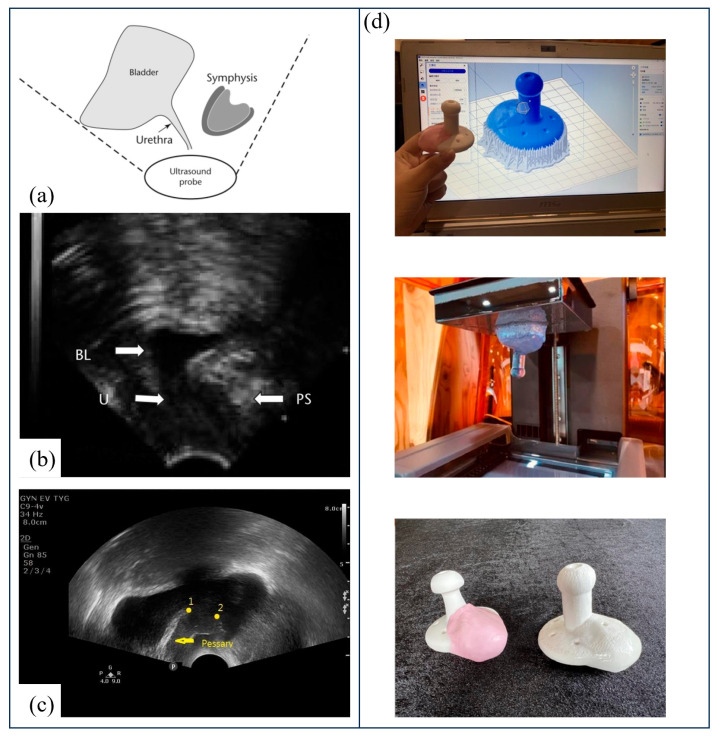
The workflow for customizing vaginal pessaries using 3D printing. (**a**) Transvaginal ultrasound focusing on the retropubic region, including urethra, paraurethral tissue, and bladder. (**b**) Representative ultrasound image showing anatomical landmarks (BL, bladder; U, urethra; PS, pubic symphysis). (**c**) Ultrasound-guided positioning of the Gellhorn pessary (arrow); the distance between points 1 and 2 was assumed as an ellipsoid to estimate the unsupported gap. (**d**) Subsequent digital modeling and 3D printing process, with representative images of the final customized pessary [59].

To address this gap, Barsky et al. reported the first successful use of a patient-specific 3D-printed pessary [58]. Using FDM and medical-grade silicone, the authors demonstrated the feasibility of producing customized devices when commercial products fail to fit. This proof-of-concept emphasized the transformative role of additive manufacturing in tailoring pessaries to patient anatomy. Building on this concept, Lin et al. developed a clinically applicable workflow that integrates transvaginal ultrasound, Otoform silicone impressions, and 3D printing to create tailor-made pessaries for women with advanced POP [60]. As illustrated in Figure 7a–c, ultrasound was first used to identify anatomical gaps not supported by a standard Gellhorn pessary, and Otoform molds were combined with the pessary to generate patient-specific templates. These were then digitized and converted into 3D models for printing. The final devices, produced with medical-grade silicone, are shown in Figure 7d. In a pilot trial of six women, the customized pessaries were well tolerated and led to significant improvements in validated quality-of-life scores, with no adverse effects such as discomfort, expulsion, or erosion. Hong et al. further demonstrated the feasibility of patient-specific pessaries by employing 3D-printed molds filled with biocompatible silicone rubber [61]. In a pilot trial with eight women, the patient-specific pessaries significantly improved prolapse-related symptoms and satisfaction compared with standard devices, underscoring the clinical utility of additive manufacturing for personalized pessary design. Beyond improving anatomical fit, innovation has also extended to therapeutic function. Long et al. reported the development of an estriol-eluting pessary using 3D-printed molds, which achieved sustained local hormone release for over three months while maintaining comparable mechanical strength to commercial devices [62]. This drug delivery approach offers the dual benefit of structural support and the mitigation of vaginal atrophy, a frequent comorbidity in POP patients. More broadly, Sethi and Yadav highlighted in their narrative review that 3D-printed, patient-specific, and drug-releasing pessaries represent promising directions to improve long-term adherence and patient autonomy, addressing the persistent challenges of fitting, comfort, and self-management [59].

Together, these studies demonstrate how 3D printing can overcome the intrinsic limitations of traditional pessaries by enabling truly individualized design, improving anatomical fit, and enhancing patient autonomy in POP management.

### 2.3. Imaging and Surgical Planning Tools

Clinically, POP is most commonly evaluated using the Pelvic Organ Prolapse Quantification (POP-Q) system, which relies on physical examination and anatomical landmarks to stage prolapse severity [1]. Although POP-Q remains the gold standard, it is inherently examiner-dependent, requires in-clinic assessment, and provides limited capability for patient self-monitoring or longitudinal evaluation outside specialized settings. These limitations have prompted growing interest in complementary diagnostic approaches that may improve accessibility, reproducibility, and patient engagement, thereby potentially facilitating earlier detection and more continuous follow-up.

**Figure 8:**
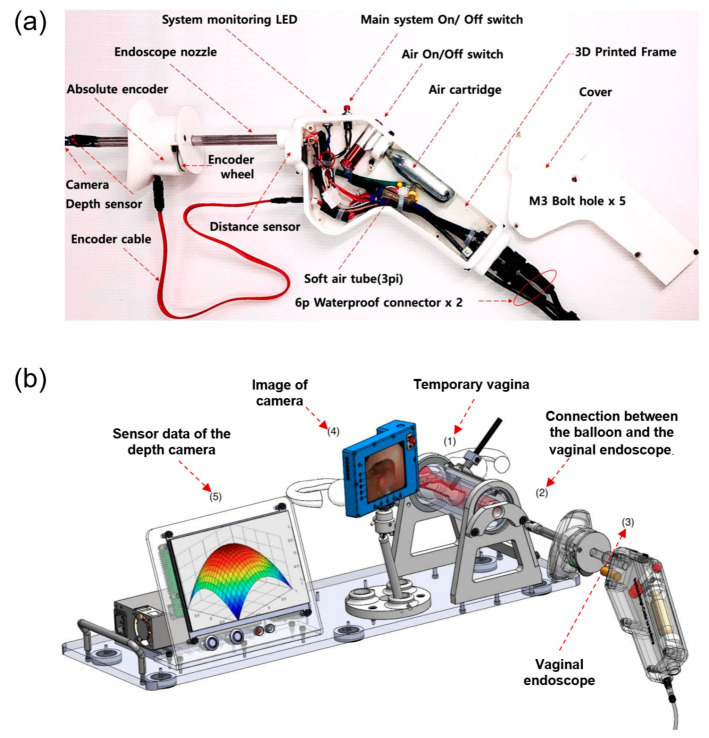
A representative example of a vaginal endoscope prototype enabled by three-dimensional (3D) printing for exploratory self-assessment of pelvic organ prolapse. (**a**) Computer-aided design and mechanical configuration of the endoscope fabricated using additive manufacturing, illustrating the integration of structural components and internal features. (**b**) Bench-top feasibility testing of the 3D-printed prototype in a simulated environment, demonstrating functional assembly and proof-of-concept evaluation [65].

Beyond implant fabrication, early applications of three-dimensional (3D) printing in POP have primarily focused on enabling the rapid prototyping of novel diagnostic and assessment devices. In an exploratory study by Jun et al. [63], 3D printing was employed as a fabrication platform to develop a vaginal endoscope intended for POP self-assessment. Additive manufacturing enabled the integration of complex internal geometries, sensor housings, and inflatable components within a compact device architecture, thereby demonstrating the feasibility of producing customized gynecological instruments without reliance on conventional tooling [64]. Figure 8 illustrates the design and testing of the vaginal endoscope prototype: panel (a) shows the computer-aided design (CAD) model and mechanical configuration, highlighting the integration of structural components and internal functional features; panel (b) presents the bench-top feasibility test in a simulated environment, demonstrating functional assembly and proof-of-concept validation.

Compared with traditional fabrication techniques such as injection molding or subtractive machining, 3D printing offers distinct advantages for early-stage medical device development, particularly in terms of design flexibility, geometric complexity, and cost-effectiveness for low-volume production [66]. Conventional manufacturing typically requires dedicated molds or extensive post-processing, which can limit rapid iteration and substantially increase development time and cost [65]. In contrast, additive manufacturing allows direct translation of digital designs into physical prototypes, facilitating rapid design modification, functional integration, and small-batch fabrication. These characteristics make 3D printing especially well suited for the development of patient-oriented POP assessment devices, where iterative optimization, anatomical customization, and proof-of-concept validation are essential.

Importantly, the contribution of these studies lies not in establishing a new diagnostic standard for POP, but in demonstrating how 3D printing can support early-stage device development, iterative design optimization, and proof-of-concept validation for patient-oriented assessment tools. Although the clinical utility of such endoscopic systems remains to be rigorously validated, these works exemplify how additive manufacturing may lower technical barriers to innovation in POP diagnostics by enabling flexible design, rapid modification, and small-batch fabrication of specialized devices.

## 3. Conclusions & Perspectives

POP remains a prevalent and clinically challenging condition, particularly in aging populations, where both conservative and surgical management strategies are constrained by limited personalization, suboptimal biomechanical compatibility, and concerns regarding long-term safety. In this context, three-dimensional (3D) printing has emerged as a transformative technology with the capacity to address several of the fundamental limitations associated with conventional pelvic floor devices. For clarity and accessibility, the main application domains of 3D printing in POP management, including common material systems, key requirements, current validation levels, and major limitations, are summarized in Table 1.

This narrative review summarizes recent progress in the application of 3D printing for POP management, focusing on three principal domains: (i) the fabrication of next-generation meshes for pelvic floor repair, (ii) the development of patient-specific vaginal pessaries, and (iii) imaging-assisted models and devices for surgical planning, education, and exploratory self-assessment. Collectively, the reviewed studies demonstrate that additive manufacturing enables unprecedented control over geometry, mechanical compliance, and material composition, allowing pelvic floor implants and devices to be tailored to individual anatomical and functional requirements.

Among implantable solutions, 3D-printed meshes based on biodegradable polymers such as polycaprolactone, elastomeric systems including thermoplastic polyurethanes and polycarbonate urethanes, natural biomaterials such as silk fibroin, and hydrogel-based constructs such as poly (vinyl alcohol) represent a clear departure from traditional polypropylene meshes. By decoupling mechanical performance from material chemistry through architectural design, these systems can more closely reproduce the nonlinear, compliant behavior of vaginal tissue while simultaneously improving tissue integration and reducing chronic inflammatory responses. Moreover, the compatibility of 3D printing with composite formulations and drug incorporation highlights the potential for multifunctional meshes that combine mechanical reinforcement with localized therapeutic delivery. Clinically, these developments should be interpreted in comparison with existing standards of POP repair. While conventional polypropylene meshes offer durable support, their fixed material properties and mesh architecture may contribute to complications such as poor tissue conformity, erosion, and chronic inflammatory responses. By contrast, 3D-printed meshes may allow closer matching to pelvic floor biomechanics through patient-specific design and tunable architecture. Nevertheless, whether these engineering advantages translate into superior clinical outcomes over native tissue repair or currently available mesh systems remains to be established.

In parallel, the application of 3D printing to customized vaginal pessaries illustrates how additive manufacturing can directly impact conservative POP management. Patient-specific designs derived from imaging or physical impressions have demonstrated improved anatomical fit, comfort, and short-term clinical outcomes compared with standardized commercial devices. The integration of drug-eluting functions further suggests a pathway toward combined mechanical and pharmacological therapy, which may improve long-term adherence and quality of life in selected patient populations. Clinically, the value of these innovations becomes clearer when compared with conventional pessaries, which are generally limited to standardized shapes and sizes and often require repeated fitting adjustments. By contrast, 3D-printed pessaries may offer superior anatomical conformity and individualized support, potentially improving comfort, retention, and adherence. Nevertheless, these proposed advantages still require confirmation in larger studies directly comparing customized devices with commercially available pessaries in terms of safety, durability, usability, and patient-reported outcomes.

Beyond therapeutic devices, 3D printing–enabled workflows have also shown value in imaging analysis, anatomical modeling, and surgical planning. Although many of these applications remain at the proof-of-concept stage, they exemplify how digital reconstruction and rapid prototyping can support individualized assessment, preoperative decision-making, and medical education, potentially complementing established clinical evaluation systems such as POP-Q.

Despite these promising advances, several challenges must be addressed before the widespread clinical translation of 3D-printed POP devices can be achieved. Current evidence remains dominated by preclinical studies and small pilot trials, with limited large-scale, long-term clinical data directly comparing 3D-printed devices with established standards of care. Beyond clinical validation, regulatory translation represents a major real-world hurdle. Compared with conventional off-the-shelf products, 3D-printed and patient-specific devices introduce additional complexity in regulatory classification, manufacturing consistency, and quality assurance, making existing approval pathways difficult to apply directly. Clearer regulatory frameworks for customized implants, pessaries, and other personalized devices will therefore be essential. At the same time, greater standardization is needed across the entire development pipeline, including material characterization, printing parameters, sterilization procedures, mechanical testing, biological safety evaluation, and batch-to-batch reproducibility. Post-market surveillance will also be critical, particularly for implanted or long-term indwelling devices, to monitor complications such as erosion, deformation, material aging, degradation, device failure, and patient-reported outcomes under real-world conditions. Finally, the cost-effectiveness and scalability of these technologies must be carefully evaluated. Progress in this field will depend not only on technological innovation, but also on closer alignment between engineering advances, regulatory science, and clinical governance.

In conclusion, 3D printing offers a versatile and powerful platform for advancing personalized management of pelvic organ prolapse across surgical, conservative, and diagnostic domains. While its clinical adoption is still in an early phase, continued interdisciplinary research and well-designed translational studies are likely to position additive manufacturing as a key enabling technology in the next generation of urogynecological devices and patient-centered POP care.

## Figures and Tables

**Table 1:** Overview of main application domains of 3D printing in POP management.

Application Domain	Common Materials/Systems	Main Clinical/Technical Value	Current Evidence
Meshes	PCL, TPU/PCU, silk fibroin, PVA hydrogels	Better biomechanical matching, tunable architecture, improved biocompatibility, drug-loading potential	Predominantly preclinical
Vaginal pessaries	Medical-grade silicone, silicone-based molds, drug-eluting systems	Personalized fit, improved comfort, better retention, conservative individualized therapy	Early pilot clinical evidence
Imaging and planning tools	Imaging-derived digital/printed models, prototype devices	Surgical planning, education, rapid prototyping, individualized assessment	Proof-of-concept/technical validation

## Data Availability

The original contributions presented in the study are included in the article; further inquiries can be directed to the corresponding author.
